# Office Notes

**Published:** 1871-06

**Authors:** W. H. Shadoan


### OFFICE NOTES.

BY W. H. SHADOAN, D. D. S.

(Continued from page 105.)

To attach sectional block teeth to silver plate, use Watt & Williams' metal. Grind and arrange the teeth the same as for rubber. Invest in Watt's moulding flask, the same as for their process. After the flask is separated, remove the plate and polish the surface that the attachments are intended to take hold of, coat it with jewelers' liquid solder, or deliquessed chloride of zinc, and dip the plate into a portion of the melted metal, and return to its place and cast same as where the whole plate is to be of that material. On removing the case from the investment, the teeth will be found firmly united to the plate. Care should be taken that no more fullness be had to the wax than is absolutely necessary, as there will be a waste of metal as well as labor. Any one at all acquainted with the working of this metal, knows that it does not work down as easily as rubber. Teeth may be attached to aluminum in the same way, by making a sufficient number of holes through the plate under the pins in the teeth. These holes are to be about a line in diameter and countersunk on the back side, and in addition to the holes, the plate should be roughened with a graver to add to the strength of the attachment. Finish same as for rubber work.

The surface of the aluminum may be more highly finished with a warm burnisher, using parafine as a lubricator. This can be removed with chloroform, and the process repeated until a satisfactory finish is obtained. Where the teeth are to be brought close to the gum, owing to a prominence to the alveolar border, the plate need not extend beyond the shoulder of the teeth. Slit the plate a little way, about a sixteenth to an eighth of an inch apart, and with the pliers twist these, reversing the turn so as to give additional security to the hold on the aluminum. A little experience in this will be quite sufficient.

(To be continued.)

